# Bisphenol A Exposure Induces Sensory Processing Deficits in Larval Zebrafish during Neurodevelopment

**DOI:** 10.1523/ENEURO.0020-22.2022

**Published:** 2022-05-16

**Authors:** Courtney Scaramella, Joseph B. Alzagatiti, Christopher Creighton, Samandeep Mankatala, Fernando Licea, Gabriel M. Winter, Jasmine Emtage, Joseph R. Wisnieski, Luis Salazar, Anjum Hussain, Faith M. Lee, Asma Mammootty, Niyaza Mammootty, Andrew Aldujaili, Kristine A. Runnberg, Daniela Hernandez, Trevor Zimmerman-Thompson, Rikhil Makwana, Julien Rouvere, Zahra Tahmasebi, Gohar Zavradyan, Christopher S. Campbell, Meghna Komaranchath, Javier Carmona, Jennifer Trevitt, David Glanzman, Adam C. Roberts

**Affiliations:** 1 Department of Psychology, California State University at Fullerton, Fullerton, CA 92831; 2 Department of Molecular, Cellular, and Developmental Biology, University of California, Santa Barbara, Santa Barbara, CA 93106; 3 Department of Biology, California Institute of Technology, Pasadena, CA 91125; 4 Department of Neuroscience, University of California, Riverside, Riverside, CA 92521; 5 Department of Society and Genetics, University of California, Los Angeles, Los Angeles, CA 90095; 6 Saint Louis University School of Medicine, St. Louis, MO 63104; 7 Washington University School of Medicine, St. Louis, MO 63110; 8 Department of Integrative Biology and Physiology, University of California, Los Angeles, Los Angeles, CA 90095; 9 Department of Molecular and Cell Biology, University of California, Berkeley, Berkeley, CA 94720; 10 Department of Neuroscience, The University of Arizona, Tucson, AZ 85719; 11 Department of Biomedical Engineering, Columbia University, New York, NY 10027; 12 Department of Physics, University of California, Los Angeles, Los Angeles, CA 90095; 13 Department of Neurobiology, David Geffen School of Medicine at University of California, Los Angeles, Los Angeles, CA 90095; 14 Integrative Center for Learning and Memory, Brain Research Institute, David Geffen School of Medicine at University of California, Los Angeles, Los Angeles, CA 90095

**Keywords:** autism spectrum disorder, C-start reflex, habituation, Mauthner cell, prepulse inhibition, zebrafish

## Abstract

Because of their *ex utero* development, relatively simple nervous system, translucency, and availability of tools to investigate neural function, larval zebrafish are an exceptional model for understanding neurodevelopmental disorders and the consequences of environmental toxins. Furthermore, early in development, zebrafish larvae easily absorb chemicals from water, a significant advantage over methods required to expose developing organisms to chemical agents *in utero*. Bisphenol A (BPA) and BPA analogs are ubiquitous environmental toxins with known molecular consequences. All humans have measurable quantities of BPA in their bodies. Most concerning, the level of BPA exposure is correlated with neurodevelopmental difficulties in people. Given the importance of understanding the health-related effects of this common toxin, we have exploited the experimental advantages of the larval zebrafish model system to investigate the behavioral and anatomic effects of BPA exposure. We discovered that BPA exposure early in development leads to deficits in the processing of sensory information, as indicated by BPA’s effects on prepulse inhibition (PPI) and short-term habituation (STH) of the C-start reflex. We observed no changes in locomotion, thigmotaxis, and repetitive behaviors (circling). Despite changes in sensory processing, we detected no regional or whole-brain volume changes. Our results show that early BPA exposure can induce sensory processing deficits, as revealed by alterations in simple behaviors that are mediated by a well-defined neural circuit.

## Significance Statement

Bisphenol A (BPA) exposure elicits sensory processing deficits in larval zebrafish. Specifically, animals show abnormal prepulse inhibition (PPI) and short-term habituation (STH), which are behaviors mediated by a relatively simple neural circuit: the C-start escape circuit. Given the well-defined nature of the circuitry underlying the C-start reflex, the present study should facilitate future investigations of the neurobiological basis of BPA-induced behavioral deficits. Furthermore, the behavioral assays used here can be readily adapted for high-throughput screening of potential therapeutic agents. Finally, the present study provides a model system and a set of assays that can generally be used to investigate sensory processing, locomotion, anxiety, and key anatomic measurements.

## Introduction

Bisphenol A (BPA) and BPA analogs are common chemicals found in polycarbonate plastics and other products. They are also used to help extend the shelf life of food products (Vandenberg et al., 2007; Chen et al., 2016). Because of the ubiquitous nature of BPA-containing products, measurable levels of BPA and BPA analogs are found in human breast milk, blood, and cerebral spinal fluid (Ye et al., 2006; Vandenberg et al., 2007; Stein et al., 2015). Fetal plasma and amniotic fluid contain detectable levels of BPA, indicating that it can cross the maternal-fetal-placental barrier and influence fetal development (Schonfelder et al., 2002; Yamada et al., 2002). Thus, BPA can potentially impact human development *in utero* and postnatally.

BPA exposure is associated with human disease and dysfunction, including fertility problems, cancer, obesity, and neurobehavioral abnormalities (Rochester, 2013). In particular, BPA exposure is correlated with the development of neurodevelopmental disorders, such as autism spectrum disorder (ASD), and children diagnosed with ASD have higher BPA metabolites in their urine (de Cock et al., 2012; Stein et al., 2015; Yoo et al., 2020; Hansen et al., 2021). Further, multiple studies have shown that behavioral problems correlate with maternal BPA exposure (Ejaredar et al., 2017).

BPA can potentially disrupt cellular function through multiple molecular mechanisms, including disruption of endocrine function and chromosomal modifications. BPA perturbs endocrine function and can bind to several endocrine-related receptors, such as estrogen receptors (Mustieles et al., 2015). BPA exposure changes gene expression by modulating transcription factors and perturbing the epigenome (Dolinoy et al., 2007; Yaoi et al., 2008; Lam et al., 2011; Singh and Li, 2012), the latter effect can have transgenerational consequences (Santangeli et al., 2019). Finally, BPA disrupts double-stranded DNA repair mechanisms and increases oxidative stress (Iso et al., 2006; Allard and Colaiácovo, 2010; Durovcova et al., 2018)

Rodents have been used extensively to model human neurodevelopmental disorders, such as ASD. The molecular perturbations underlying rodent models of ASD result in social deficits, repetitive behaviors, and cognitive inflexibility (Moy et al., 2006; Ellegood and Crawley, 2015; Kazdoba et al., 2016). In mice, prenatal BPA exposure elicits several ASD-like phenotypes, including increased anxiety, repetitive behaviors, sensory processing difficulties, and impairments in memory and social behaviors. The impairments observed in mice exposed to BPA suggest that this toxin might contribute to human ASD or related neurodevelopmental difficulties (Wolstenholme et al., 2013; Hu et al., 2022).

Modeling human BPA exposure in animals is difficult because the half-life of BPA is ∼6 h, and these measurements only represent a snapshot of exposure (Thayer et al., 2015). Human samples do not provide information regarding the range of BPA exposure or the impact of exposure to the toxin at critical periods of human development. Understanding the developmental consequences of BPA exposure, which is known to modify DNA methylation, is particularly important because a single application of a DNA methyltransferase inhibitor in mice at postnatal day (P)7 induces life-long behavioral abnormalities and deficits in synaptic plasticity (Subbanna et al., 2016).

Unlike the complications involved in exposing common laboratory mammals to toxins because of their *in utero* development, chemical agents can be efficiently and effectively delivered to embryonic and larval zebrafish *ex utero*. It has been found that exposure to relatively high concentrations of BPA early in the zebrafish life cycle increases mortality and deformity, and delays hatching. Interestingly, despite general agreement regarding the adverse effects of high levels of BPA on mortality, body shape, and time of hatching, the reported threshold concentration of BPA for producing these effects varies greatly (Lam et al., 2011; Saili et al., 2012; Tse et al., 2013; Wang et al., 2013; Martínez et al., 2018; Olsvik et al., 2019; Coumailleau et al., 2020; Gyimah et al., 2021; Huang et al., 2021; Scopel et al., 2021; Sundarraj et al., 2021; Wu et al., 2021). Furthermore, some studies have found that BPA exposure modifies locomotion in zebrafish, whereas others have not (Saili et al., 2012; Wang et al., 2013; Kinch et al., 2015; Fraser et al., 2017; Olsvik et al., 2019; Coumailleau et al., 2020; Gyimah et al., 2021; Wu et al., 2021). Given the complexity of the underlying mechanisms of behavior, it is not surprising that BPA exposure can produce widely disparate behavioral results. Nonetheless, exposing larval zebrafish to BPA clearly modifies neural circuitry involved in locomotion and, therefore, BPA exposure during development would be expected to cause behavioral alterations. Here, we investigated the consequences of early BPA exposure on sensory processing, locomotion, and anxiety in zebrafish larvae, as well as on anatomy.

## Materials and Methods

### Animals

The TL strain of zebrafish was used for all experiments except for those in which brain volume was measured. For the measurements of brain volume, we used transgenic zebrafish that express the photoconvertible protein Kaede pan-neuronally Tg(elavl3:Kaede)RW0130a (RRID: ZFIN ID: ZDB-GENO-060619–2; Sato et al., 2006).

### BPA exposure

After zebrafish eggs were collected, they were placed in E3 water (5 mm NaCl, 0.33 mm MgSO_4_, 0.33 mm CaCl_2_, 0.17 mm KCl, 1 mm HEPES, 10^−5^% methylene blue, pH 7.0) and transferred to the laboratory for staging. Embryos were staged to ∼4–5 h after fertilization; they were then placed in Petri dishes containing E3 water alone, E3 water containing 0.05% dimethyl sulfoxide (DMSO), or E3 water containing BPA (10–50 μm) in 0.05% DMSO, which were put into an incubator (14/10 h light/dark; 28.5°C). Each day, the dishes were cleaned by replacing the solutions in the Petri dishes, and any dead embryos were removed and counted. On the fifth day of exposure, all solutions were replaced with E3, and the larvae were maintained in E3 until testing at 6 d postfertilization (dpf). On testing day, fish were fed premixed food (Hatchfry Encapsulon) in E3. This food was removed 4 h later, the solution in the Petri dishes was replaced with fresh E3, and then experimental protocols were begun (≥1 h after the removal of the food).

### Analyses of survival and deformity

In experiments designed to determine minimum BPA concentrations that caused death, deformities, or hatching delays, we assayed the effect of varying concentrations of BPA on these variables. By the end of the sixth day, the total number of fish that survived was calculated for each plate of embryos to determine mortality (0 = alive at 6 dpf, 1 = dead at 6 dpf). Concurrently, deformities of the surviving embryos were recorded (0 = no deformity at 6 dpf, 1 = deformity at 6 dpf) and photographed. Common deformities included spinal/tail bends, cranial malformations, and yolk and pericardial edema. Failure to escape the chorion by 6 dpf was also classified as a deformity. Animals with deformities were euthanized on 6 dpf and were not used for behavioral experiments. Finally, to determine whether hatching was delayed (Kimmel et al., 1995), the day the embryos escaped from their chorions was recorded (1 = hatched after 3 dpf or failed to hatch, 0 = hatched before 3 dpf).

### Behavioral protocols

#### Audiogenic startle

To measure the C-start reflex, surviving and nondeformed zebrafish larvae (6 dpf) were placed into individual wells with a 1.63-cm diameter (∼3 ml of E3) in a 24-well plate. The plate was then positioned on a lightbox (Gagne Inc.) next to a speaker. The volume of the speaker was calibrated in air with a decibel meter (re 20 μPa). After a 30-min period of acclimation, six auditory/vibrational (AV) pulses (200 Hz, 108 dB re 20 μPa, 2-ms pulse duration) were delivered every 5 min. A high-speed camera (Mako U-029B; Allied Vision) positioned above the plate was used to record (500 fr/s) the responses of the larvae to the AV pulse. The determination of whether or not an AV pulse elicited a C-start was made by visual inspection of the video recording by a blind observer. Long-latency startle responses (≥26 ms) were not counted as C-starts; such long-latency startles are mediated by different neurocircuitry than are the shorter latency C-starts (Liu and Fetcho, 1999; Burgess and Granato, 2007; Kohashi and Oda, 2008).

#### Prepulse inhibition (PPI)

Zebrafish larvae (6 dpf) were placed into individual wells (see above) and allowed to acclimate for 30 min. After this acclimation period, six AV stimuli were delivered (5-min intertrial interval), and C-start reflexes were recorded and quantified as described above. AV stimuli were alternatively delivered with or without a prepulse auditory stimulus. The prepulse stimulus was 100 ms in duration (200 Hz, 55 dB re 20 μPa) and preceded the startle stimulus (2-ms duration, 500 Hz, 108 dB re 20 μPa) by 100 ms. The relative difference in startles with and without the prepulse stimulus was used to calculate the PPI index [(percentage startle response without the prepulse stimulus – percent startle response with the prepulse stimulus)/(the average startle response without a prepulse)].

#### Short-term habituation (STH)

To measure STH, larvae (6 dpf) were placed into individual wells in a 24-well plate as described above. After 30 min of acclimation, 120 AV pulses (200 Hz, 2-ms duration, 108 dB) were delivered at 1 Hz, followed by a posttest AV stimulus 30 s after the last training stimulus. The responses to the training and posttest pulses were video recorded, and only short-latency (C-start) responses were counted for the determination of habituation.

#### Thigmotaxis

Twenty zebrafish larvae (6 dpf) were put into a Petri dish (142 mm in diameter) containing ∼100-ml E3; the dish was placed inside a custom-made testing chamber with overhead illumination. The larvae were given 30 min to acclimate, after which a camera (Casio Exilim EX-ZR1000, EX-ZR800, or EX-ZR100; Casio America) was used to record a single image of the positions of all 20 fish in relation to the center and edge of the Petri dish. Using ImageJ (RRID:SCR_003070; Schneider et al., 2012), an average plate position for the fish was determined from the image; for statistical analyses, each plate average equaled an *n* of 1.

#### Locomotion

Zebrafish larvae (6 dpf) were placed individually into a Petri dish (36 mm in diameter) containing ∼8-ml E3. After 1 min, the movement of the fish was recorded for 20 min (50 f/s) by a camera (Casio Exilim EX-ZR1000, EX-ZR800, or EX-ZR100; Casio America) positioned above the Petri dish. The position of each fish during the 20-min observation period was sampled every 41.67 ms and the total distance (cm) the fish moved was calculated using Behavioral Cloud software. Also, the path taken by each fish was determined by sampling the fish’s position every 1 s. Using ImageJ, a blind observer then counted the total number of circles made by the fish during the 20-min period; this number was used as a measure of repetitive behavior (circling). Movements were considered to constitute a circle if the path taken by a fish followed a circular trajectory. A circle-like movement was only counted if the path was 75% complete (closed) before a change in direction. Additionally, circles that included more than half of the dish were not included in the circle count because they were difficult to distinguish from mere swimming in a circular enclosure (the dish).

### Anatomical measurements

#### Head size

**Figure 1.**
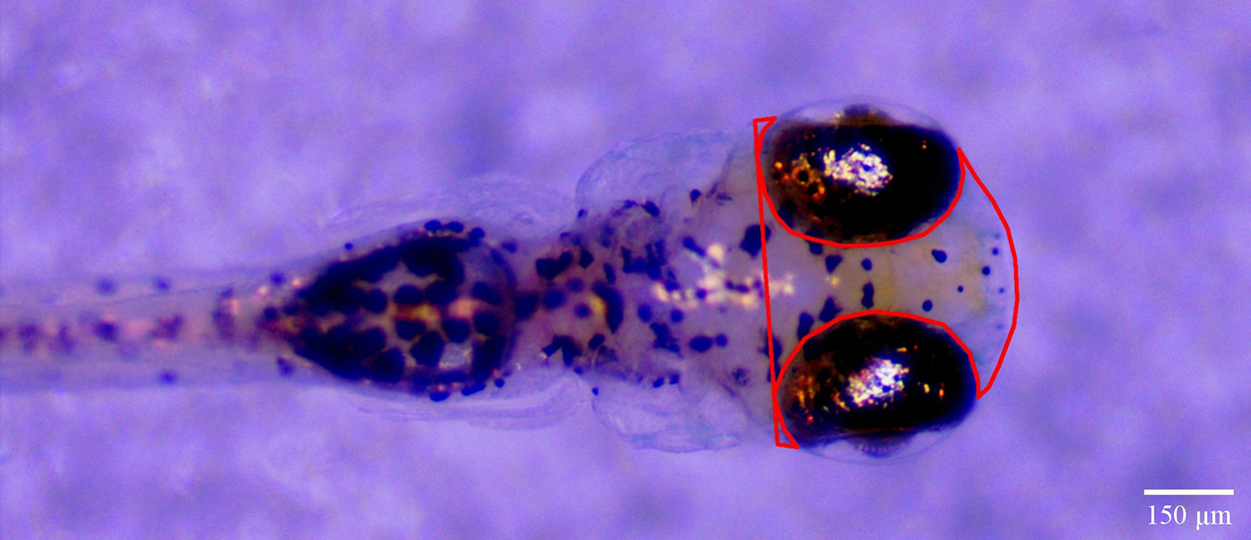
Image of a zebrafish larva illustrating the area used for head size measurements. Dorsal view of a zebrafish larvae embedded in low melting point agarose. Red lines outline the area measured to determine the head size of larvae. Scale bar: 150 μm.

Zebrafish (6 dpf) were put into tricaine (MS-222; 200 mg/l) and then positioned dorsal side up in 1% low melting point agarose. The fish were then imaged using an AmScope stereo microscope and photographed with an attached digital camera. ImageJ was used to determine the area of the dorsal portion of the head in the photographs by a blind observer. The head was outlined, starting posterior to the eyes, as indicated in Figure 1.

#### Brain size

Images of zebrafish (6 dpf) expressing the photoconvertible protein Kaede pan-neuronally driven by the ELAV3 promoter (Sato et al., 2006) were used to determine the whole-brain volume and volumes of brain regions in zebrafish exposed to BPA. To image zebrafish brains, an LSM Pascal microscope (Zeiss) was used (488-nm excitation) to construct a whole-brain image stack using z-stacks (2-μm slices) and tiling. The tiles were stitched together after the images were taken. In addition, we used depth-dependent adaptive illumination to ensure adequate detection of deeper brain regions. The calculated volumes from the 2-μm slices were then summed to determine the total volume for each region (forebrain, midbrain, and hindbrain), and these values were then added to quantify the total brain volume.

### Pharmacology

All compounds were obtained from Sigma-Aldrich. BPA (CAS 80-05-7) was dissolved in DMSO (CAS 67-68-5) to make a final concentration of 0.05%.

### Statistical analyses

Group comparisons were determined by unpaired *t* tests or ANOVAs. When appropriate, Tukey’s HSD tests were used for *post hoc* analyses. Table 1 includes a summary of the statistical analyses.

**Table 1:** Statistical analyses

	Data structure	Type of test	Power (α = 0.05)
a(Fig. 2*A*)	Non-normally distributed	One-way ANOVA test	0.92
b(Fig. 2*B*)	Non-normally distributed	One-way ANOVA test	1.00
c(Fig. 2*C*)	Non-normally distributed	One-way ANOVA test	1.00
d(Fig. 2*D*)	Normally distributed	One-way ANOVA test	0.09
e(Fig. 3*A*)	Normally distributed	Unpaired *t* test	0.07
f(Fig. 3*B*)	Non-normally distributed	Unpaired *t* test	0.19
g(Fig. 3*C*)	Normally distributed	Unpaired *t* test	0.30
h(Fig. 4*A*)	Non-normally distributed	Unpaired *t* test	0.17
i(Fig. 4*B*)	Non-normally distributed	Unpaired *t* test	0.63
j(Fig. 4*C*)	Normally distributed	Unpaired *t* test	0.07
k(Fig. 5)	Normally distributed	Unpaired *t* test	0.63
l(Fig. 6*A*)	Normally distributed	Two-way ANOVA test (interaction)	1.00
m(Fig. 6*B*)	Normally distributed	One-way ANOVA test	1.00
n(Fig. 6*C*)	Non-normally distributed	One-way ANOVA test	0.79
o(Fig. 7)	Normally distributed	Unpaired *t* test	0.12
p(Fig. 8*A*)	Normally distributed	Unpaired *t* test	0.06
q(Fig. 8*B*)	Normally distributed	Unpaired *t* test	0.30
r(Fig. 9)	Normally distributed	Unpaired *t* test	0.36
s(Fig. 10*B*)	Normally distributed	Two-way ANOVA test (interaction)	0.05
t(Fig. 10*B*)	Normally distributed	Two-way ANOVA test (main effect)	0.06
u(Fig. 10*C*)	Normally distributed	Unpaired *t* test	0.06

## Results

### Exposure to high levels of BPA increases mortality and deformity in larval zebrafish

**Figure 2.**
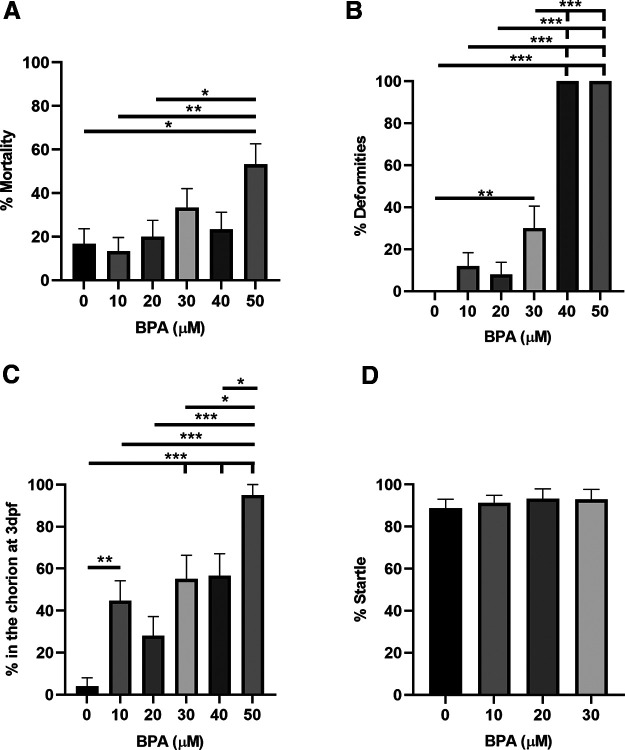
BPA exposure can increase mortality, deformity, and delays in hatching in zebrafish. ***A***, Five days of exposure to BPA increased mortality measured at 6 dpf according to a one-way ANOVA (*F*_(5,174)_ = 3.56; *p *<* *0.01). Tukey’s HSD *post hoc* tests revealed that the group exposed to 50 μm BPA had significantly (*p *<* *0.05) more mortality compared with the groups exposed to 0, 10, and 20 μm BPA. ***B***, BPA exposure (5 d) increased the number of deformities measured at 6 dpf as indicated by a one-way ANOVA (*F*_(5,126)_ = 63.41; *p *<* *0.001). Subsequent Tukey’s HSD *post hoc* tests demonstrated that the 40 and 50 μm groups had significantly (*p *<* *0.001) more deformities than did the groups exposed to 0, 10, 20, and 30 μm BPA. Furthermore, the 30 μm BPA group had significantly *(p *<* *0.01) more deformities than did the 0 μm BPA group. ***C***, BPA exposure (5 d) caused hatching delays as indicated by a one-way ANOVA (*F*_(5,136)_ = 11.48; *p *<* *0.001). Tukey’s HSD *post hoc* tests revealed that hatching in the group exposed to 50 μm BPA was significantly (*p *<* *0.05) more likely to be delayed compared with hatching in the 0, 10, 20, 30, and 40 μm BPA groups. Furthermore, the fish in the 10 and 30 μm BPA groups demonstrated significantly (*p *<* *0.01) more frequent delayed hatching than did those in the 0 μm BPA group. ***D***, Zebrafish exposed to BPA (5 d), and with no visible deformities, did not exhibit deficits in the C-start reflex as shown by a one-way ANOVA (*F*_(3,80)_ = 0.23; *p *=* *0.87). This and subsequent figures present means ± SEM; * specifies a significant difference between groups in this and subsequent figures.

**Figure 3.**
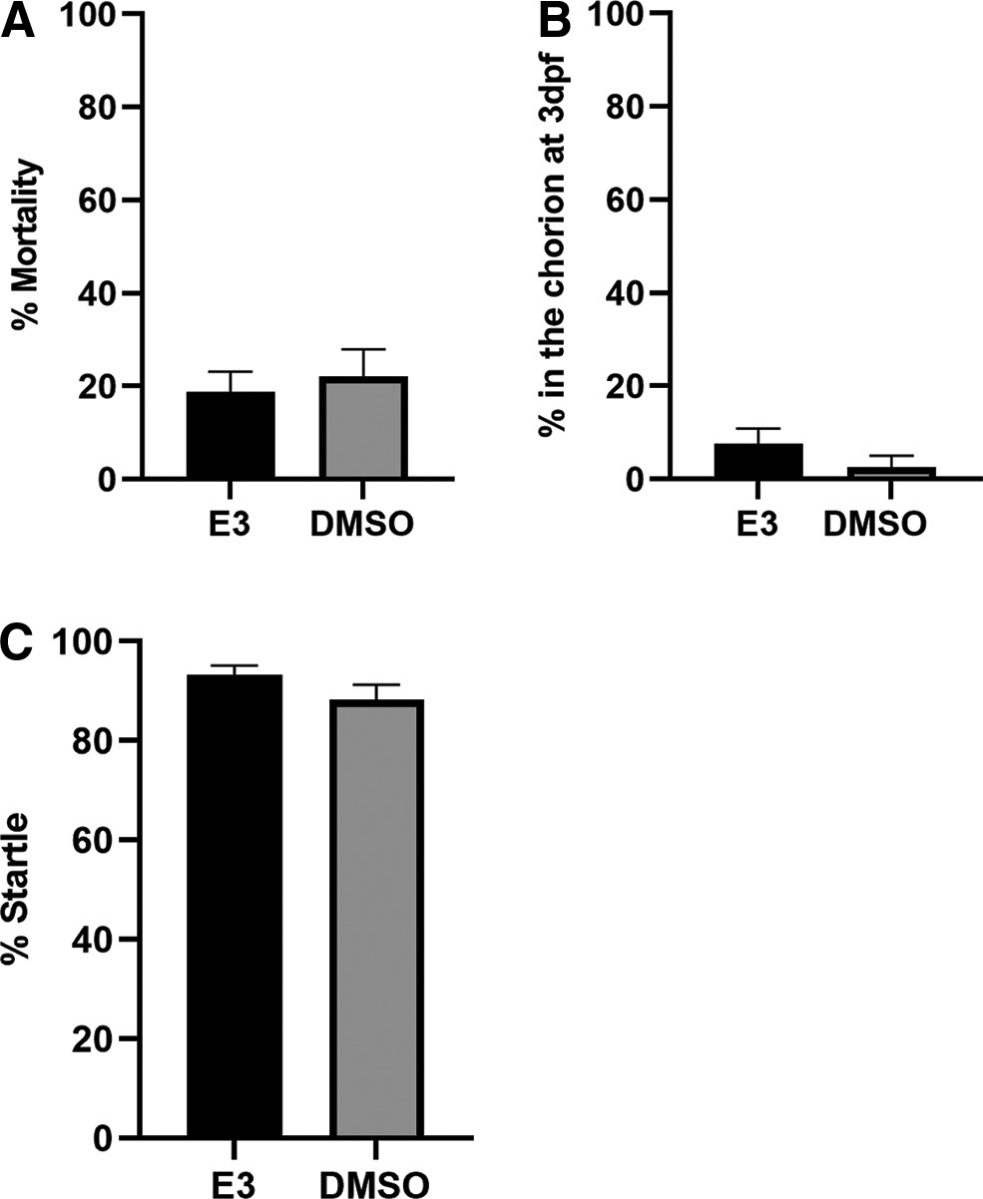
Extended exposure to DMSO does not increase mortality, delays in hatching, or abnormal sensorimotor responses. ***A***, Exposure of zebrafish larvae to DMSO (0.05%) from 0 to 5 dpf did not change the mortality rate (DMSO group = 22.00 ± 5.92%, *n *=* *50) compared with a control group exposed only to E3 (E3 group = 18.75 ± 4.39%, *n *=* *80) according to an unpaired *t* test (*t*_(128)_ = 0.45, *p *=* *0.66). ***B***, Extended exposure (5 d) to DMSO (0.05%) did not significantly delay hatching in zebrafish compared with fish exposed to E3 solution (DMSO group = 2.50 ± 2.50%, *n *=* *40; E3 group = 7.58 ± 3.28%, *n *=* *66; unpaired *t* test; *t*_(104)_ = 1.09, *p *=* *0.28). ***C***, An unpaired *t* test (*t*_(104)_ = 1.45, *p *=* *0.15) revealed no significant differences in startle response probability between the DMSO group (88.33 ± 3.00%, *n *=* *40) and the E3 group (93.18 ± 1.87%, *n *=* *66).

To determine the highest dose of BPA exposure that does not elicit increased mortality and deformity, or reduced sensorimotor responsiveness, we exposed zebrafish larvae to several doses of BPA for 5 d (Fig. 2). DMSO (0.05%) was used as the vehicle control and was included in the control group’s solutions for all experiments investigating BPA exposure. Extended exposure (5 d) to E3 or DMSO (0.05%) alone did not significantly alter the rate of larval mortality (Fig. 3*A*) or deformity [data not shown; no deformities were observed for either the E3 group (0/66) or DMSO group (0/40)]. Furthermore, there were no hatching delays (Fig. 3*B*) or reductions in sensorimotor responsiveness, as measured by startle to an auditory stimulus (Fig. 3*C*), in either the E3 or DMSO groups. High concentrations (50 μm) of BPA, however, increased mortality (Fig. 2*A*; Table 2). There was also an increased likelihood of deformity with levels of BPA ≥30 μm (Fig. 2*B*; Table 2). Relatively low concentrations (10 μm) of BPA significantly delayed hatching, as did higher concentrations (≥30 μm; Fig. 2*C*; Table 2), this effect might indicate a neurodevelopmental delay or changes to the chorion that make escaping the chorion more difficult. No significant reductions in audiogenic startle rate were observed with BPA concentrations ≤30 μm (Fig. 2*D*; Table 2). Animals with visually detectable deformities were not used to test for startle responses. Consequently, no zebrafish larvae exposed to concentrations of BPA ≥40 μm were tested for startle.

**Table 2:** Mortality, deformity, hatching delays, startle response in zebrafish larvae exposed to BPA

BPA	Mortality; mean and SEM	Deformity; mean and SEM	Hatching delays; mean and SEM	Startle response; mean and SEM
0 μm	16.67 ± 6.92% *n *=* *30	0.00 ± 0.00% *n *=* *25	4.00 ± 4.00% *n *=* *25	88.67 ± 4.38% *n *=* *25
10 μm	13.33 ± 6.31% *n *=* *30	11.54 ± 6.39% *n *=* *26	44.83 ± 9.40% *n *=* *29	91.30 ± 3.61% *n *=* *23
20 μm	20.00 ± 7.43% *n *=* *30	8.33 ± 5.76% *n *=* *24	28.00 ± 9.17% *n *=* *25	93.18 ± 4.74% *n *=* *22
30 μm	33.33 ± 8.75% *n *=* *30	30.00 ± 10.51% *n *=* *20	55.00 ± 11.41% *n *=* *20	92.86 ± 4.85% *n *=* *14
40 μm	23.33 ± 7.85% *n *=* *30	100.00 ± 0.00% *n *=* *23	56.52 ± 10.57% *n *=* *23	
50 μm	53.33 ± 9.26% *n *=* *30	100.00 ± 0.00% *n *=* *14	95.00 ± 5.00% *n *=* *20	

**Figure 4.**
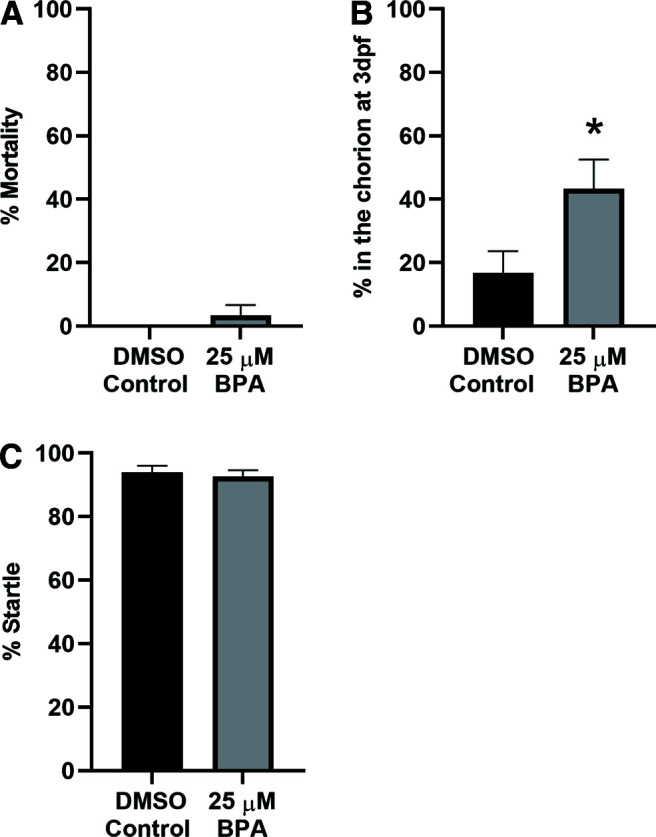
Exposure to 25 μm BPA does not alter mortality, or startle probability in zebrafish larvae, but does delay hatching. ***A***, Bathing fish in BPA for 5 d did not significantly increase mortality (BPA group, *n *=* *30) compared with bathing them in the vehicle (DMSO group, *n *=* *30; unpaired *t* test; *t*_(58)_ = 1.00, *p *=* *0.32). ***B***, BPA treatment significantly delayed hatching (BPA group, *n *=* *30; DMSO group, *n *=* *30; unpaired *t* test, *t*_(58)_ = 2.32, *p *=* *0.02). ***C***, The startle probability of BPA-treated larvae (BPA group, *n *=* *29) did not differ from that of vehicle-treated larvae (DMSO group, *n *=* *30; unpaired *t* test; *t*_(57)_ = 0.45, *p *=* *0.66).

A recent report by Scopel et al. (2021) investigated the effect of 25 μm BPA on mortality, deformity, and hatching in larval zebrafish. Because Scopel et al. (2021) only exposed zebrafish to 25 μm BPA for 3 d, we investigated the consequences of a 5-d exposure of larvae to 25 μm BPA. As shown in Figure 4*A*, larvae exposed to 25 μm BPA did not exhibit a statistically significant increase in mortality (BPA group = 3.33 ± 3.33) compared with the vehicle control group (DMSO group = 0.00 ± 0.00). Furthermore, no deformities were observed in either the BPA group (*n *=* *29) or the DMSO control group (*n *=* *30; data not shown). We did detect (Fig. 4*B*) a significant delay in hatching in the BPA group (43.33 ± 9.20) compared with the DMSO group (16.67 ± 6.92). With respect to the percent evoked startle response, there were no significant differences observed between the BPA group (92.53 ± 2.12) and the DMSO group (93.89 ± 2.19; Fig. 4*C*). Based on these findings, 25 μm was selected as the experimental concentration to investigate the behavioral and anatomic effects of BPA exposure from 0 to 5 dpf.

### BPA exposure disrupts sensorimotor gating in zebrafish larvae

**Figure 5.**
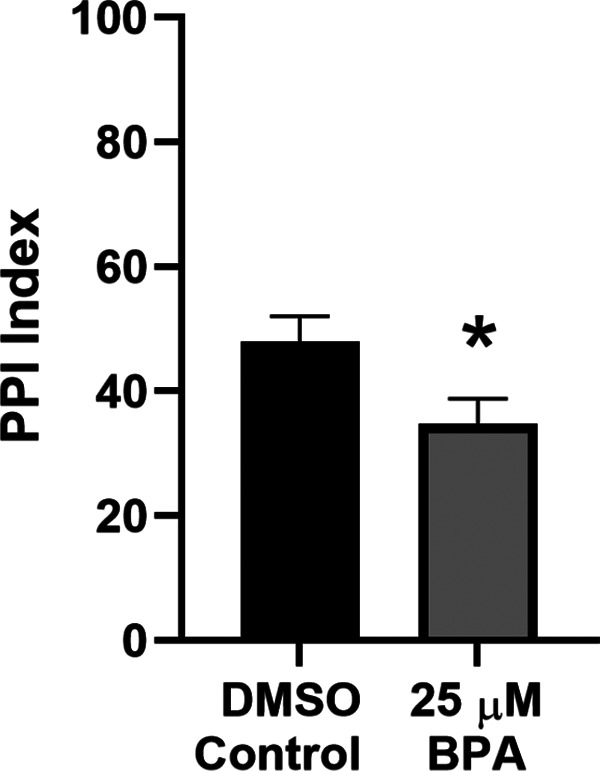
BPA exposure disrupts PPI in zebrafish larvae. Effect on PPI of exposure to 25 μm BPA for 5 d (BPA group, *n *=* *83) compared with that of exposure to the vehicle (DMSO group, *n *=* *83; unpaired *t* test, *t*_(164)_ = 2.31, *p *<* *0.05).

As measured by PPI, deficits in sensorimotor gating have been observed in humans with neurodevelopmental disorders, such as schizophrenia and ASD (Braff et al., 1978, 2001; Grillon et al., 1992; McAlonan et al., 2002; Frankland et al., 2004; Perry et al., 2007; Kohl et al., 2013; but see Ornitz et al., 1993; Madsen et al., 2014). Further, animal models of these developmental disorders also display deficits in PPI (Dulawa et al., 1997; Swerdlow and Geyer, 1998; Burgess and Granato, 2007; Bickel et al., 2008; Möhrle et al., 2021). To determine whether BPA exposure causes deficits in PPI, we treated zebrafish with 25 μm BPA from 0 to 5 dpf. At 6 dpf, we gave BPA-treated (experimental) and DMSO-treated (control) larvae brief auditory stimuli to elicit a C-start, either with or without a prepulse stimulus. We calculated the level of PPI for experimental and control zebrafish. As shown in Figure 5, prolonged exposure to 25 μm BPA significantly reduced PPI (BPA group = 34.74 ± 4.03) compared with vehicle-treated controls (DMSO group = 47.99 ± 4.08). Thus, BPA exposure appears to affect the neural circuitry mediating sensorimotor gating.

### BPA exposure disrupts STH in zebrafish larvae

**Figure 6.**
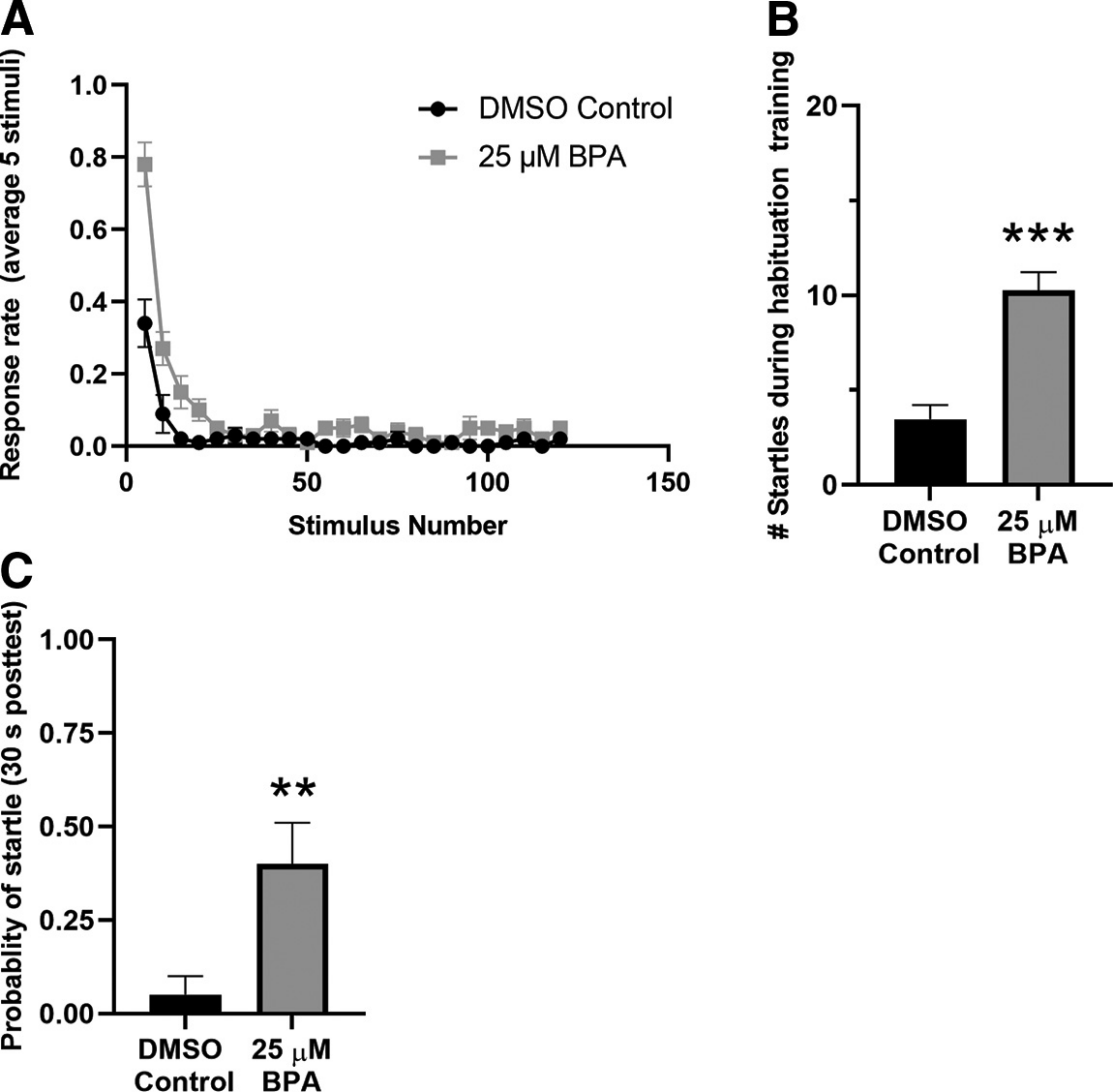
BPA exposure reduces STH. ***A***, Response rates during habituation training of BPA-exposed fish (*n *=* *20) and DMSO-treated fish (*n *=* *20). Data were binned as a running average of five consecutive auditory pulses. According to a two-way ANOVA, the number of responses during training and the probability of startle at 30 s posttest produced a significant interaction (*F*_(1,76)_ = 27.24; *p *<* *0.01). ***B***, Results of a one-way ANOVA, subsequent to the two-way ANOVA in ***A***, comparing the number of startle responses during habituation training by the BPA-treated group to the DMSO-treated group. This analysis revealed that the BPA group habituated less than did the DMSO group (*F*_(1,38)_ = 30.58; *p *<* *0.001). ***C***, Results of a one-way ANOVA comparing the number of startle responses evoked on the 30 s posttest in the BPA-treated and DMSO-treated groups (*F*_(1,38)_ = 8.10; *p *<* *0.01).

Neurodevelopmental disorders exhibit cognitive difficulties, including deficits in habituation, suggesting problems processing sensory information (Barry and James, 1988; Bolino et al., 1992, 1994; Braff et al., 1992; Martineau et al., 1992; Vivanti et al., 2018; Bharath et al., 2020; Gandhi et al., 2021; Jamal et al., 2021). Similar to humans with neurodevelopmental disorders, mouse models of neurodevelopmental disorders also exhibit reductions in habituation (Dulawa et al., 1997; Restivo et al., 2005; Bickel et al., 2008; Lovelace et al., 2016; Möhrle et al., 2021). The extensive literature on habituation in zebrafish larvae (Eaton et al., 1977; Roberts et al., 2011, 2013, 2016, 2019; Wolman et al., 2011) makes this model organism particularly well suited for investigating abnormal habituation resulting from neurodevelopmental disorders. To test the effect of chronic exposure to BPA on STH in zebrafish larvae, we gave larvae 120 AV pulses (1 Hz). There was significantly less habituation (more startle responses) during training in BPA-treated larvae (BPA group = 10.25 ± 0.98 startles) than in the control group (DMSO group = 3.45 ± 0.11; Fig. 6*A*,*B*). The BPA-treated group also showed significantly less habituation (higher probability of startle) on a posttest taken 30 s after the end of habituation training (BPA group = 0.40 ± 0.11) than did the control group (DMSO group = 0.05 ± 0.05; Fig. 6*C*).

### Thigmotaxis was not altered by exposure to BPA

**Figure 7.**
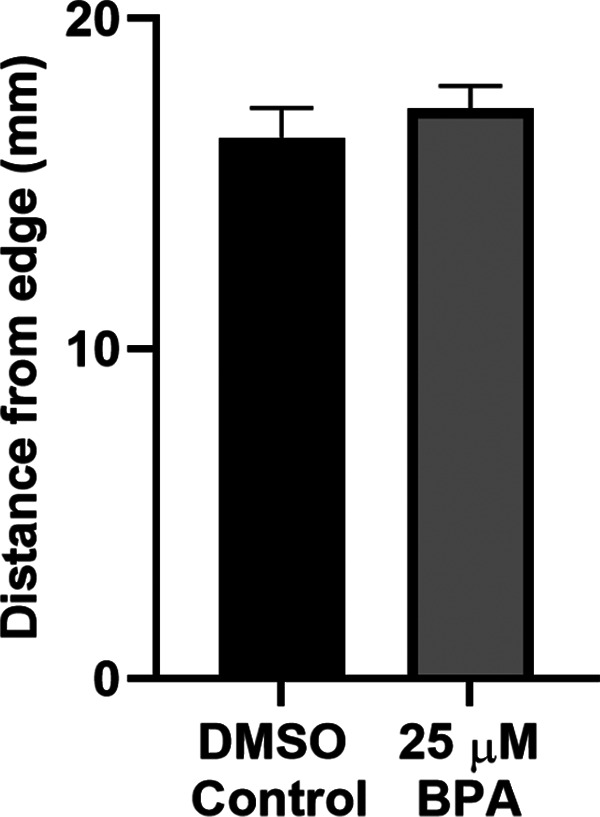
Thigmotaxis was not increased in zebrafish exposed to BPA. The mean distance from the edge of the experimental dish during swimming in the BPA-exposed group (*n *=* *14) and the DMSO-treated group (*n *=* *15; *t* test, *t*_(27)_ = 0.77, *p *=* *0.45).

Organisms tend to move toward the edges of an open area when anxious (thigmotaxis), and their position in space can serve as a measurement of anxiety (Christmas and Maxwell, 1970; Prut and Belzung, 2003; Schnörr et al., 2012; Ahmad and Richardson, 2013; Walz et al., 2016). To assess whether 5 d of exposure to BPA affects thigmotaxis in zebrafish, we quantified their propensity to thigmotax when put into a Petri dish (142 mm in diameter). As shown in Figure 7, we did not observe a significant difference between BPA-treated zebrafish (BPA group = 17.27 ± 0.69 mm) and control zebrafish (DMSO group = 16.40 ± 0.89 mm) with respect to their distance from the dish’s edge. Thus, BPA exposure did not increase anxiety in zebrafish, at least as measured by this assay.

### BPA exposure did not modify locomotion in zebrafish larvae

**Figure 8.**
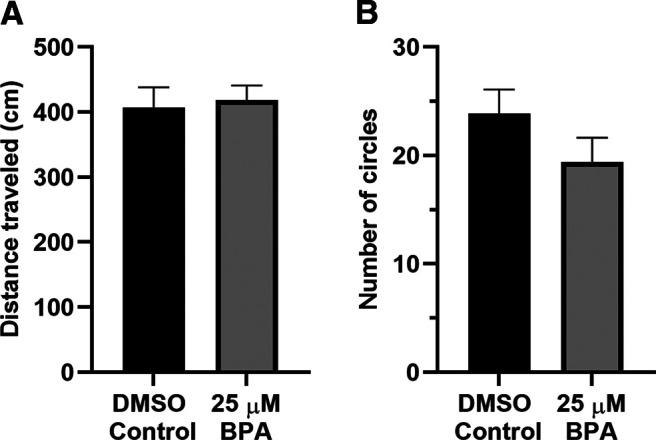
BPA exposure did not modify locomotion in zebrafish larvae. ***A***, Mean distance traveled by BPA-exposed (*n *=* *32) and DMSO-exposed (*n *=* *31) fish (unpaired *t* test; *t*_(61)_ = 0.30, *p *=* *0.77). ***B***, Number of circles made by larvae exposed to BPA (BPA group, *n *=* *32) and the vehicle (DMSO group, *n *=* *31; *t*_(61)_ = 1.44, *p *=* *0.15).

In contrast to the results of Saili et al. (2012), we did not find that BPA exposure increased locomotion. The BPA group traveled 418.86 ± 22.33 cm during the 20-min test period, whereas the control (DMSO) group traveled 407.33 ± 30.62 cm (Fig. 8*A*). We also tested whether BPA exposure increased the propensity for larvae to perform repetitive behaviors (Dwivedi et al., 2019). For this test, we quantified the number of circles made by zebrafish larvae during the same 20-min test period (Fig. 8*B*). The number of circles made during swimming by the BPA-treated group (19.41 ± 2.22) was not different from the number made by the DSMO-treated group (23.90 ± 2.18; Fig. 8*B*). Thus, BPA exposure did not produce any significant changes in this repetitive behavior.

### Head size and brain volume were unchanged in zebrafish larvae by BPA exposure

**Figure 9.**
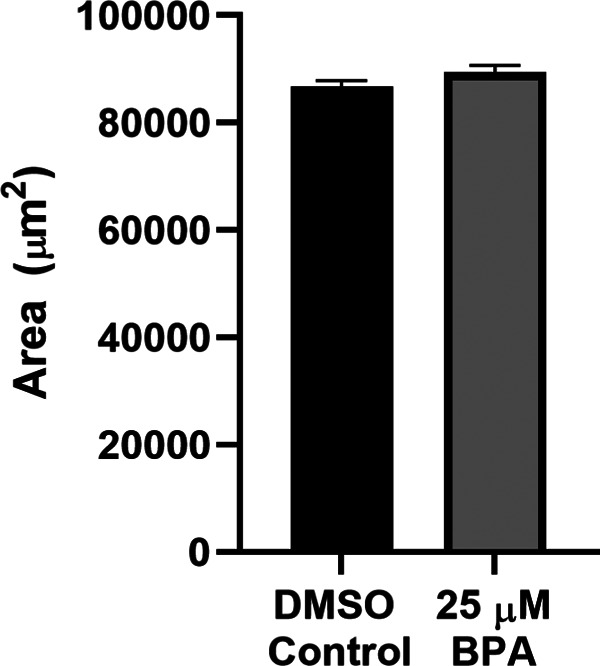
Exposure to BPA did not change head size in zebrafish larvae. Mean volume of the head in BPA-treated (BPA group, *n *=* *30) and vehicle-treated (DMSO group, *n *=* *30) fish (*t*_(58)_ = 1.63, *p *=* *0.11).

We wished to know whether BPA exposure affects head size in zebrafish larvae. Accordingly, we measured the area of the dorsal portion of the head, as indicated in Figure 1. We found no difference in head size between a group of zebrafish exposed to BPA for 5 d (BPA group = 89,491.07 ± 1231.97μm^2^) and a control group (DMSO group = 86,742.87 ± 1145.39 μm^2^; Fig. 9). Thus, our BPA treatment did not affect larval head size.

**Figure 10.**
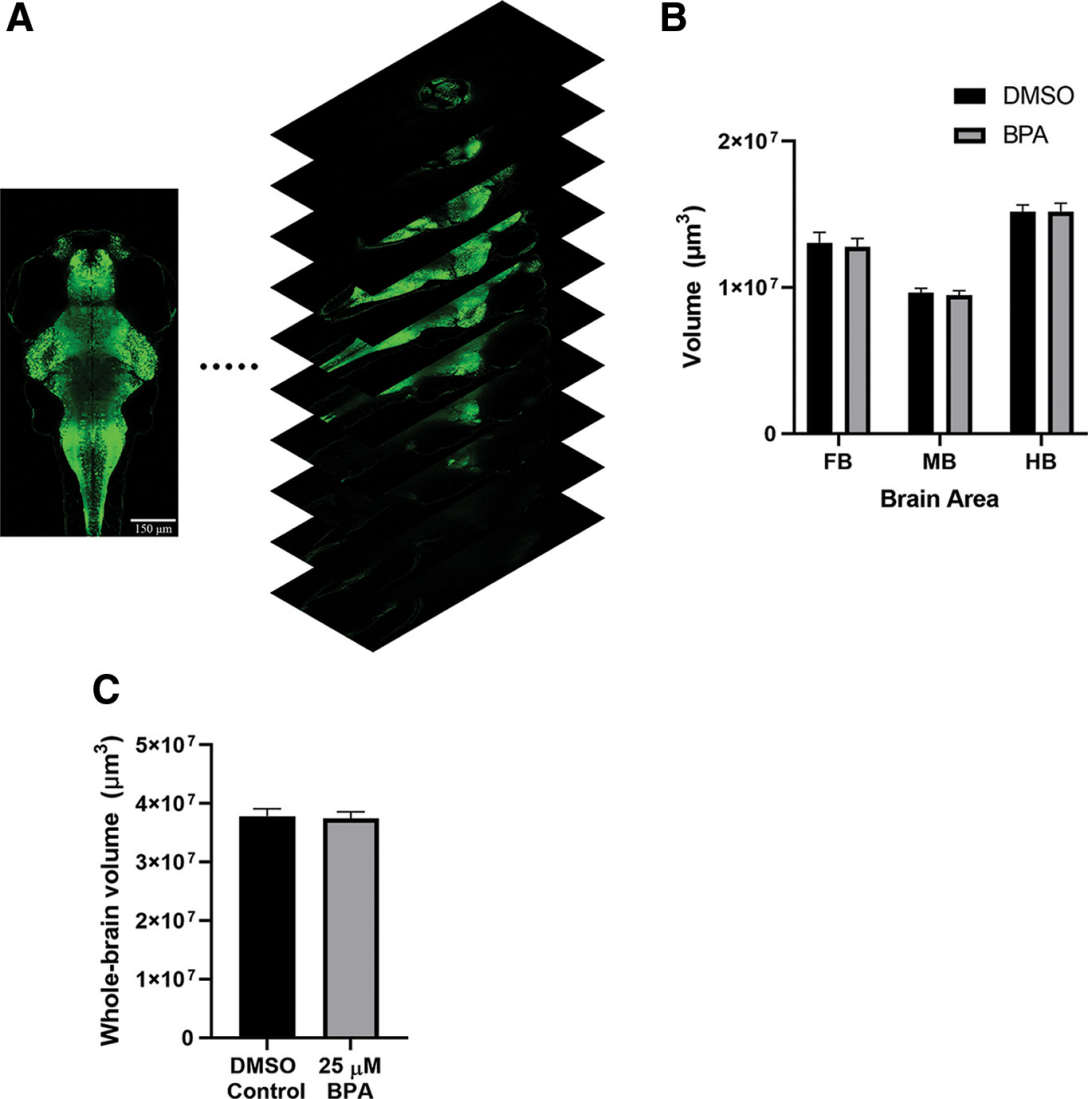
Brain volume was not changed in zebrafish larvae by BPA exposure. ***A***, 3D reconstruction of a larval brain. Sample confocal images of optical sections of a brain exposed to DMSO. Scale bar: 150 μm. ***B***, Mean volumes of the forebrain (FB), midbrain (MB), and hindbrain (HB) in BPA-exposed (BPA group, *n *=* *15) fish compared with the vehicle-exposed fish (DMSO group, *n *=* *15). A two-way ANOVA revealed no significant interaction or group effect (interaction, *F*_(2,84)_ = 0.03; *p *=* *0.75: group, *F*_(1,84)_ = 0.10; *p *=* *0.97. ***C***, Mean volume of the whole brain in BPA-exposed fish (BPA group, *n *=* *15) and vehicle-exposed fish (DMSO group, *n *=* *15). The difference between the two groups was not significant (*t*_(28)_ = 0.23, *p *=* *0.82).

We also examined whether brain volume was altered in larvae exposed to BPA. Therefore, we measured the volume of the forebrain, midbrain, and hindbrain in fish treated with BPA or DMSO alone. We observed no significant differences in the volume of the forebrain (BPA group = 12,792,608.93 ± 563,489.39 μm^3^; DMSO group = 13,046,271.33 ± 722,424.18 μm^3^), midbrain (BPA group = 9,472,418.03 ± 302,444.26 μm^3^; DMSO group = 9,618,611.83 ± 324,266.03 μm^3^), and hindbrain (BPA group = 15,168,202.24 ± 594,253.33 μm^3^; DMSO group = 15,168,013.01 ± 468,219.30 μm^3^; Fig. 10*A*). Additionally, we summed these brain area volumes to determine whole-brain volume in the larvae. The whole-brain volume did not differ significantly in the BPA-treated (37,433,229.20 ± 1,133,165.488) and the vehicle-treated (DMSO = 37,832,896.17 ± 1,281,757.20) groups (Fig. 10*B*).

## Discussion

Here, we examined the effect of early exposure to BPA, an environmental toxin, on development in the zebrafish. To date, most investigations of the behavioral ramifications of BPA exposure have focused on locomotion; there has been little emphasis on sensory processing despite evidence that humans and animal models of neurodevelopmental disorders commonly involve disruptions of sensory processing, including PPI and habituation (Braff et al., 1978, 1992; Barry and James, 1988; Bolino et al., 1992, 1994; Grillon et al., 1992; Martineau et al., 1992; Dulawa et al., 1997; Swerdlow and Geyer, 1998; Braff et al., 2001; McAlonan et al., 2002; Frankland et al., 2004; Restivo et al., 2005; Burgess and Granato, 2007; Perry et al., 2007; Bickel et al., 2008; Kohl et al., 2013; Lovelace et al., 2016; Vivanti et al., 2018; Bharath et al., 2020; Gandhi et al., 2021; Jamal et al., 2021; Möhrle et al., 2021; but see Ornitz et al., 1993; Madsen et al., 2014). Both PPI and habituation test the ability of an organism to ignore unnecessary sensory information. We found reductions in PPI and habituation (during and 30 s after training) of the C-start reflex in larvae exposed to BPA (Figs. 5, 6). Because the neural circuitry and molecules underlying these two behavioral phenomena are well-described in larval zebrafish (Liu and Fetcho, 1999; Burgess and Granato, 2007; Kohashi and Oda, 2008; Roberts et al., 2011, 2019; Wolman et al., 2011; Kohashi et al., 2012; Bergeron et al., 2015; Marsden and Granato, 2015; Wolman et al., 2015; Tabor et al., 2018; Nelson et al., 2020; Zoodsma et al., 2020), elucidation of the BPA-induced pathology should be relatively straightforward. Furthermore, habituation and PPI of the C-start reflex are behaviors that are well-suited for high-throughput drug screening, which should facilitate the development of novel treatments to mitigate the effects of BPA (Rihel et al., 2010; Thyme et al., 2019).

We found that 25 μm BPA was the highest level of exposure (5 d) that did not result in an increase in gross deformities and significant mortality (Figs. 2, 4). Generally, this finding is consistent with the literature; however, the exact level of BPA to elicit these effects varies greatly (Lam et al., 2011; Saili et al., 2012; Tse et al., 2013; Wang et al., 2013; Martínez et al., 2018; Olsvik et al., 2019; Coumailleau et al., 2020; Gyimah et al., 2021; Huang et al., 2021; Scopel et al., 2021; Sundarraj et al., 2021; Wu et al., 2021). The differences in the reported concentrations of BPA required to elicit deformity and mortality likely result from variation in experimental protocols or the strain of zebrafish used. Similar to deformity and mortality, hatching delays have more commonly been reported with higher levels of BPA (Martínez et al., 2018; Scopel et al., 2021; but see Olsvik et al., 2019 compared with lower levels of BPA (Gyimah et al., 2021; Huang et al., 2021; Gu et al., 2022; but see Sundarraj et al., 2021). By contrast, very low levels of BPA have been reported to accelerate hatching (Qiu et al., 2016; Coumailleau et al., 2020). We observed hatching delays at BPA concentrations as low as 10 μm, as well as at higher concentrations (Figs. 2*C*, 4*B*). Changes to the chorion might be responsible for the observed delays; however, we think it is more likely that hatching latencies stem from delays in neural or skeletomuscular development. Notice that early BPA exposure in our study did not diminish the probability of startle (C-start reflex), which suggests that the BPA-treated zebrafish were not in poor health at the time of testing (Figs. 2*D*, 4*C*).

In humans, neurodevelopmental disorders such as ASD are often comorbid with anxiety (Lai et al., 2019), animal models of ASD also tend to exhibit comorbidity with anxiety, as indicated in such tasks as the open field test in mice (Hung et al., 2008; Blundell et al., 2009; Schmeisser et al., 2012; Clipperton-Allen and Page, 2014). In 5-dpf zebrafish larvae, Fraser et al. (2017) found that BPA exposure (10 μm) increased anxiety, as measured by thigmotaxis. By contrast, we did not detect a change in thigmotaxis despite using a higher concentration of BPA (25 μm; Fig. 7). While several experimental parameters (see below) differed between our experiments and those of Fraser et al. (2017), they measured the effect of BPA exposure while the toxin was present at the time of testing, whereas BPA was not present during behavioral testing in our experiments. Therefore, the enhanced thigmotaxis observed by Fraser et al., may be an acute effect of BPA. In addition, we used a larger behavioral chamber than did Fraser et al. (2017). Furthermore, our measure of thigmotaxis was the position of the zebrafish as a continuous variable (distance from the edge; Roberts et al., 2020) rather than the level of activity within a defined zone in the chamber (Schnörr et al., 2012). The larger chamber combined with a continuous variable should have increased the likelihood of detecting baseline thigmotaxis compared with alternative methods. As such, we think the different concentrations of BPA or exposure models most likely account for the different results. Consequently, we believe that the differences between our results and those of previous studies of BPA’s effects on thigmotaxis in zebrafish can be ascribed to differences in experimental variables, including differences in the concentrations of BPA used.

Numerous studies have found that BPA affects locomotion in zebrafish. However, there is little consistency in reported behavioral outcomes (Saili et al., 2012; Wang et al., 2013; Kinch et al., 2015; Fraser et al., 2017; Olsvik et al., 2019; Coumailleau et al., 2020; Gyimah et al., 2021; Wu et al., 2021). We observed no changes in the total distance moved or in repetitive behavior (circling; Fig. 8) in BPA-exposed fish. Possibly, the disparities in results of the studies on locomotion can be attributed to methodological differences. Fraser et al. (2017) observed that the presence or absence of a light cycle and the size of the testing chamber could substantially influence BPA’s effect on locomotion. Fraser and colleagues also claimed that BPA-induced hyperlocomotion requires dark rearing, and our larvae were reared with a day/night cycle.

BPA has been reported to modify neural structure and function in mice (Inadera, 2015) and to increase neurogenesis in the hypothalamus of zebrafish (Kinch et al., 2015). Studies of BPA’s effect on neurogenesis throughout the central nervous system have yielded conflicting reports (Gyimah et al., 2021; Gu et al., 2022). We found no BPA-related change in the volume of the forebrain, midbrain, or hindbrain, or in total brain volume (Fig. 10). This suggests that the PPI and habituation deficits could not have resulted from any gross changes in brain morphology. However, our results do not preclude the possibility of more subtle BPA-induced modifications in neural morphology.

Model developmental systems such as larval zebrafish, which permit in-depth analyses of toxin-induced molecular, cellular, and behavioral changes, are important for understanding how chronic exposure to environmental toxins such as BPA disrupts neural development and behavior. The behavioral assays used in our study revealed the disruptive effects of BPA exposure on sensory processing and learning in zebrafish larvae. Our finding that both PPI and STH of the C-start are abnormal in BPA-exposed larvae should facilitate the identification of the neurobiological causes of the perturbations of sensory processing caused by early BPA exposure.

## Synthesis

Reviewing Editor: Matthew Grubb, King’s College London Decisions are customarily a result of the Reviewing Editor and the peer reviewers coming together and discussing their recommendations until a consensus is reached. When revisions are invited, a fact-based synthesis statement explaining their decision and outlining what is needed to prepare a revision will be listed below. The following reviewer(s) agreed to reveal their identity: NONE.

Both Reviewers believe the study provides useful information for the field.

After consultation and discussion, our highest priority is for the discrepancy of thigmotaxis assay results between the current study and previously reported findings to be addressed, both experimentally and with clear discussion in the manuscript. If different ages, BPA administration protocols, behavioural measures and/or well sizes might account for the observed discrepancy, these factors should be investigated wherever possible to provide clarity for the field.

The authors should also address each of the Reviewers’ specific comments listed below. Note, however, that we do *not* expect any additional investigations into the neural circuitry or mechanisms underlying the observed effects.

Reviewer 1 comments:

This is a toxicological study in which the authors examine the effects of Bisphenol A, a well-studied toxin, on locomotor activity and startle escape response of embryonic zebrafish. BPA has been implicated in numerous neurobiological disorders the mechanisms of which remain poorly understood. Therefore, findings of this study would be of broad interest. The authors show that high concentrations of BPA lead to high mortality and deformities rates and delays in hatching. At lower BPA dosage (25uM), the authors show a disruption of the animals’ startle response illustrated in elevated sensitivity to Pre-pulse stimuli and differences in habituation rates to repeated auditory stimuli compared to controls. It’s argued that BPA’s effects are limited to the c-start circuit because swimming activity is unaffected. Based on large scale map of embryonic brains exposed to BPA, the authors argue that the effects of BPA are not due to gross morphological changes in the brain morphology.

The manuscript is well written, and the results are clearly illustrated, I have some concerns and suggestions I wish for the authors to address with a revised version. With no particular order of importance:

1. No clear rationale stated as to why PPI is an appropriate assay to examine disruption in sensory processing. It’s sort of implied. It’s well known that ASD patients show PPI deficits manifested in persistent hypersensitivity to sound. I imagine that’s the authors’ motivation of using PPI as a test but it’s not obvious to the reader.

2. If the authors’ claim is correct that BPA disrupts auditory processing leading to a heightened sensitivity to sound in BPA exposed embryos, Then I’m curious as to why the authors did not test the animals’ auditory escape responses to increasing sound decibels ranging from 55-108dB. Would not that provide a better measurement to test disruption in sensitivity compared to the PPI protocol? The predication would be that at lower dB auditory pulses the probability of C-start response of embryos incubated in BPA would be higher compared to DMSO incubated embryos. As a guide, I refer the authors to (figure 1C) in Neumeister H et. al. Social and ecological regulation of a decision-making circuit. J Neurophysiol. 2010 Dec;104(6):3180-8.

3. Methods: There is no mention of how sound pressure amplitudes of pulses were calibrated. Would you clarify how loudness of auditory pulses were calibrated? I imagine that calibration was done in air vs. the water chambers!! If so, then a notation of re 20 μPa should be included in the methods. If the authors used a hydrophone, then a re 1 μPa notation would be appropriate.

4.PPI protocol: any particular reason why the pre-pulse frequency (200Hz) differed from the pulse stimulus (500HZ)?

5.Line 51: I think it should read “double-stranded” instead of “double-strand"

6.The central finding of the paper is that BPA exposure leads to deficits in sensory processing, while locomotor activity remains intact. Although the data supports the interpretation, What I find missing is where within the sensory components of the sensory circuit of the startle response BPA is affecting: is it the auditory hairs, the VIIIth nerve, the Mauthner command cell? I wish for the authors to elaborate on that however briefly in their discussion.

7.Figure 6A: it would be nice to increase the time resolution to observe the gradual decline in habituation rate in the BPA expose animals. Currently the data was binned into 10 sec bins, and the response rate tappers off rapidly. My point here is that the only obvious difference between the two conditions is the first 2 points of binned data. If the data is binned over 5 seconds, it might look more convincing. Secondly, the addition of roster plots of individual animals might provide more details of the richness and variation among animals. Just a thought!!!

8.Figure 10: what is the hypothesis being tested? It’s not clear to me. Given the earlier figures 1-4 where the authors already established the proper dosage that is not lethal nor leads to severe morphological deformities to continue with their studies, one would expect that there will not be significant differences in brain volume between control and BPA incubated embryos. In other words, the experimental embryos incubated at 25uM of BPA are the preselected group, there one would not expect to observe significant changes in brain volume.

Reviewer 2 comments:

The evaluation of BPA toxicity is an important task, given the broad occurrence of this chemical. Zebrafish are an excellent model for such testing, due to high fecundity, optical transparency for imaging, small size for hight throughput assessments and more. It is therefore not surprising that the model has been intensively been used already in connection with BPA toxicity, as also pointed out by the authors of the present study. This includes evaluations about mortality, deformations, locomotion, social interactions, cell death, axon growth up to transcriptional changes in treated animals. There is quite some inconsistency among the different studies, but I do not see that the current study adds something significantly different. Overall, I therefore think that the advance is limited.

The authors report different findings to those previously published by others regarding the thigmotaxis assay. In the discussion they speculate if the acute presence of BPA, the fact they measure a position rather than activity or the different age (5dpf vs 6 dpf) could lead to such different results. It seems to me that all these parameters would be very easily tested and could have been included in the study to present more standardised methods. For example, a common way to measure thigmotaxis is the amount of time the larva spends in proximity of the wall versus inside the well (and not just an image capture at one give time point).

Finally, the authors report differences in PPI and habituation, but do not further assess this. They further describe that the circuitry underlying these behaviors is well described and assessment therefore straightforward. I think the manuscript would be significantly improved if any of such assessments would be included.

## References

[B1] Ahmad F, Richardson MK (2013) Exploratory behaviour in the open field test adapted for larval zebrafish: impact of environmental complexity. Behav Processes 92:88–98. 10.1016/j.beproc.2012.10.014 23123970

[B2] Allard P, Colaiácovo MP (2010) Bisphenol A impairs the double-strand break repair machinery in the germline and causes chromosome abnormalities. Proc Natl Acad Sci U S A 107:20405–20410. 10.1073/pnas.1010386107 21059909 PMC2996676

[B3] Barry RJ, James AL (1988) Coding of stimulus parameters in autistic, retarded, and normal children: evidence for a two-factor theory of autism. Int J Psychophysiol 6:139–149. 10.1016/0167-8760(88)90045-1 3397316

[B4] Bergeron SA, Carrier N, Li GH, Ahn S, Burgess HA (2015) Gsx1 expression defines neurons required for prepulse inhibition. Mol Psychiatry 20:974–985. 10.1038/mp.2014.106 25224259 PMC4362800

[B5] Bharath R, Moodithaya SS, Halahalli H, Undaru SB, Nallilu SK, Mirajkar AM (2020) Evaluation of sympathetic sudomotor responses to auditory stimuli in children with autism spectrum disorders. Indian J Psychiatry 62:494–500. 10.4103/psychiatry.IndianJPsychiatry_573_19 33678829 PMC7909012

[B6] Bickel S, Lipp HP, Umbricht D (2008) Early auditory sensory processing deficits in mouse mutants with reduced NMDA receptor function. Neuropsychopharmacology 33:1680–1689. 10.1038/sj.npp.1301536 17712349

[B7] Blundell J, Tabuchi K, Bolliger MF, Blaiss CA, Brose N, Liu X, Südhof TC, Powell CM (2009) Increased anxiety-like behavior in mice lacking the inhibitory synapse cell adhesion molecule neuroligin 2. Genes Brain Behav 8:114–126. 10.1111/j.1601-183X.2008.00455.x 19016888 PMC2648807

[B8] Bolino F, Manna V, Di Cicco L, Di Michele V, Daneluzzo E, Rossi A, Casacchia M (1992) Startle reflex habituation in functional psychoses: a controlled study. Neurosci Lett 145:126–128. 10.1016/0304-3940(92)90002-O 1465206

[B9] Bolino F, Di Michele V, Di Cicco L, Manna V, Daneluzzo E, Casacchia M (1994) Sensorimotor gating and habituation evoked by electro-cutaneous stimulation in schizophrenia. Biol Psychiatry 36:670–679. 10.1016/0006-3223(94)91176-2 7880936

[B10] Braff D, Stone C, Callaway E, Geyer M, Glick I, Bali L (1978) Prestimulus effects on human startle reflex in normals and schizophrenics. Psychophysiology 15:339–343. 10.1111/j.1469-8986.1978.tb01390.x 693742

[B11] Braff DL, Grillon C, Geyer MA (1992) Gating and habituation of the startle reflex in schizophrenic patients. Arch Gen Psychiatry 49:206–215. 10.1001/archpsyc.1992.01820030038005 1567275

[B12] Braff DL, Geyer MA, Light GA, Sprock J, Perry W, Cadenhead KS, Swerdlow NR (2001) Impact of prepulse characteristics on the detection of sensorimotor gating deficits in schizophrenia. Schizophr Res 49:171–178. 10.1016/S0920-9964(00)00139-0 11343875

[B13] Burgess HA, Granato M (2007) Sensorimotor gating in larval zebrafish. J Neurosci 27:4984–4994. 10.1523/JNEUROSCI.0615-07.2007 17475807 PMC6672105

[B14] Chen D, Kannan K, Tan H, Zheng Z, Feng YL, Wu Y, Widelka M (2016) Bisphenol analogues other than BPA: environmental occurrence, human exposure, and toxicity-a review. Environ Sci Technol 50:5438–5453. 10.1021/acs.est.5b05387 27143250

[B15] Christmas AJ, Maxwell DR (1970) A comparison of the effects of some benzodiazepines and other drugs on aggressive and exploratory behaviour in mice and rats. Neuropharmacology 9:17–29. 10.1016/0028-3908(70)90044-4 5464000

[B16] Clipperton-Allen AE, Page DT (2014) Pten haploinsufficient mice show broad brain overgrowth but selective impairments in autism-relevant behavioral tests. Hum Mol Genet 23:3490–3505. 10.1093/hmg/ddu057 24497577

[B17] Coumailleau P, Trempont S, Pellegrini E, Charlier TD (2020) Impacts of bisphenol A analogues on zebrafish post-embryonic brain. J Neuroendocrinol 32:e12879. 10.1111/jne.12879 32749037

[B18] de Cock M, Maas YG, van de Bor M (2012) Does perinatal exposure to endocrine disruptors induce autism spectrum and attention deficit hyperactivity disorders? Review. Acta Paediatr 101:811–818. 10.1111/j.1651-2227.2012.02693.x 22458970

[B19] Dolinoy DC, Huang D, Jirtle RL (2007) Maternal nutrient supplementation counteracts bisphenol A-induced DNA hypomethylation in early development. Proc Natl Acad Sci U S A 104:13056–13061. 10.1073/pnas.0703739104 17670942 PMC1941790

[B20] Dulawa SC, Hen R, Scearce-Levie K, Geyer MA (1997) Serotonin1B receptor modulation of startle reactivity, habituation, and prepulse inhibition in wild-type and serotonin1B knockout mice. Psychopharmacology (Berl) 132:125–134. 10.1007/s002130050328 9266609

[B21] Durovcova I, Spackova J, Puskar M, Galova E, Sevcovicova A (2018) Bisphenol A as an environmental pollutant with dual genotoxic and DNA-protective effects. Neuro Endocrinol Lett 39:294–298. 30531698

[B22] Dwivedi S, Medishetti R, Rani R, Sevilimedu A, Kulkarni P, Yogeeswari P (2019) Larval zebrafish model for studying the effects of valproic acid on neurodevelopment: an approach towards modeling autism. J Pharmacol Toxicol Methods 95:56–65. 10.1016/j.vascn.2018.11.006 30500431

[B23] Eaton RC, Farley RD, Kimmel CB, Schabtach E (1977) Functional development in the Mauthner cell system of embryos and larvae of the zebra fish. J Neurobiol 8:151–172. 10.1002/neu.480080207 856948

[B24] Ejaredar M, Lee Y, Roberts DJ, Sauve R, Dewey D (2017) Bisphenol A exposure and children’s behavior: a systematic review. J Expo Sci Environ Epidemiol 27:175–183. 10.1038/jes.2016.8 26956939

[B25] Ellegood J, Crawley JN (2015) Behavioral and neuroanatomical phenotypes in mouse models of autism. Neurotherapeutics 12:521–533. 10.1007/s13311-015-0360-z 26036957 PMC4489953

[B26] Frankland PW, Wang Y, Rosner B, Shimizu T, Balleine BW, Dykens EM, Ornitz EM, Silva AJ (2004) Sensorimotor gating abnormalities in young males with fragile X syndrome and Fmr1-knockout mice. Mol Psychiatry 9:417–425. 10.1038/sj.mp.4001432 14981523

[B27] Fraser TWK, Khezri A, Jusdado JGH, Lewandowska-Sabat AM, Henry T, Ropstad E (2017) Toxicant induced behavioural aberrations in larval zebrafish are dependent on minor methodological alterations. Toxicol Lett 276:62–68. 10.1016/j.toxlet.2017.05.021 28529144

[B28] Gandhi TK, Tsourides K, Singhal N, Cardinaux A, Jamal W, Pantazis D, Kjelgaard M, Sinha P (2021) Autonomic and electrophysiological evidence for reduced auditory habituation in autism. J Autism Dev Disord 51:2218–2228. 10.1007/s10803-020-04636-8 32926307

[B29] Grillon C, Ameli R, Charney DS, Krystal J, Braff D (1992) Startle gating deficits occur across prepulse intensities in schizophrenic patients. Biol Psychiatry 32:939–943. 10.1016/0006-3223(92)90183-z 1467378

[B30] Gu J, Guo M, Yin X, Huang C, Qian L, Zhou L, Wang Z, Wang L, Shi L, Ji G (2022) A systematic comparison of neurotoxicity of bisphenol A and its derivatives in zebrafish. Sci Total Environ 805:150210. 10.1016/j.scitotenv.2021.150210 34534871

[B31] Gyimah E, Xu H, Dong X, Qiu X, Zhang Z, Bu Y, Akoto O (2021) Developmental neurotoxicity of low concentrations of bisphenol A and S exposure in zebrafish. Chemosphere 262:128045. 10.1016/j.chemosphere.2020.128045 33182117

[B32] Hansen JB, Bilenberg N, Timmermann CAG, Jensen RC, Frederiksen H, Andersson AM, Kyhl HB, Jensen TK (2021) Prenatal exposure to bisphenol A and autistic- and ADHD-related symptoms in children aged 2 and5 years from the Odense Child Cohort. Environ Health 20:24. 10.1186/s12940-021-00709-y 33712018 PMC7955642

[B33] Hu F, Liang W, Zhang L, Wang H, Li Z, Zhou Y (2022) Hyperactivity of basolateral amygdala mediates behavioral deficits in mice following exposure to bisphenol A and its analogue alternative. Chemosphere 287:132044. 10.1016/j.chemosphere.2021.132044 34474391

[B34] Huang W, Wang X, Zheng S, Wu R, Liu C, Wu K (2021) Effect of bisphenol A on craniofacial cartilage development in zebrafish (*Danio rerio*) embryos: a morphological study. Ecotoxicol Environ Saf 212:111991. 10.1016/j.ecoenv.2021.111991 33548570

[B35] Hung AY, Futai K, Sala C, Valtschanoff JG, Ryu J, Woodworth MA, Kidd FL, Sung CC, Miyakawa T, Bear MF, Weinberg RJ, Sheng M (2008) Smaller dendritic spines, weaker synaptic transmission, but enhanced spatial learning in mice lacking Shank1. J Neurosci 28:1697–1708. 10.1523/JNEUROSCI.3032-07.2008 18272690 PMC2633411

[B36] Inadera H (2015) Neurological effects of bisphenol A and its analogues. Int J Med Sci 12:926–936. 10.7150/ijms.13267 26664253 PMC4661290

[B37] Iso T, Watanabe T, Iwamoto T, Shimamoto A, Furuichi Y (2006) DNA damage caused by bisphenol A and estradiol through estrogenic activity. Biol Pharm Bull 29:206–210. 10.1248/bpb.29.206 16462019

[B38] Jamal W, Cardinaux A, Haskins AJ, Kjelgaard M, Sinha P (2021) Reduced sensory habituation in autism and its correlation with behavioral measures. J Autism Dev Disord 51:3153–3164. 10.1007/s10803-020-04780-1 33179147

[B39] Kazdoba TM, Leach PT, Yang M, Silverman JL, Solomon M, Crawley JN (2016) Translational mouse models of autism: advancing toward pharmacological therapeutics. Curr Top Behav Neurosci 28:1–52. 10.1007/7854_2015_5003 27305922 PMC5116923

[B40] Kimmel CB, Ballard WW, Kimmel SR, Ullmann B, Schilling TF (1995) Stages of embryonic development of the zebrafish. Dev Dyn 203:253–310. 10.1002/aja.1002030302 8589427

[B41] Kinch CD, Ibhazehiebo K, Jeong JH, Habibi HR, Kurrasch DM (2015) Low-dose exposure to bisphenol A and replacement bisphenol S induces precocious hypothalamic neurogenesis in embryonic zebrafish. Proc Natl Acad Sci U S A 112:1475–1480. 10.1073/pnas.1417731112 25583509 PMC4321238

[B42] Kohashi T, Oda Y (2008) Initiation of Mauthner- or non-Mauthner-mediated fast escape evoked by different modes of sensory input. J Neurosci 28:10641–10653. 10.1523/JNEUROSCI.1435-08.2008 18923040 PMC6671347

[B43] Kohashi T, Nakata N, Oda Y (2012) Effective sensory modality activating an escape triggering neuron switches during early development in zebrafish. J Neurosci 32:5810–5820. 10.1523/JNEUROSCI.6169-11.2012 22539843 PMC6703610

[B44] Kohl S, Heekeren K, Klosterkötter J, Kuhn J (2013) Prepulse inhibition in psychiatric disorders–apart from schizophrenia. J Psychiatr Res 47:445–452. 10.1016/j.jpsychires.2012.11.018 23287742

[B45] Lai MC, Kassee C, Besney R, Bonato S, Hull L, Mandy W, Szatmari P, Ameis SH (2019) Prevalence of co-occurring mental health diagnoses in the autism population: a systematic review and meta-analysis. Lancet Psychiatry 6:819–829. 10.1016/S2215-0366(19)30289-5 31447415

[B46] Lam SH, Hlaing MM, Zhang X, Yan C, Duan Z, Zhu L, Ung CY, Mathavan S, Ong CN, Gong Z (2011) Toxicogenomic and phenotypic analyses of bisphenol-A early-life exposure toxicity in zebrafish. PLoS One 6:e28273. 10.1371/journal.pone.0028273 22194820 PMC3237442

[B47] Liu KS, Fetcho JR (1999) Laser ablations reveal functional relationships of segmental hindbrain neurons in zebrafish. Neuron 23:325–335. 10.1016/s0896-6273(00)80783-7 10399938

[B48] Lovelace JW, Wen TH, Reinhard S, Hsu MS, Sidhu H, Ethell IM, Binder DK, Razak KA (2016) Matrix metalloproteinase-9 deletion rescues auditory evoked potential habituation deficit in a mouse model of fragile X syndrome. Neurobiol Dis 89:126–135. 10.1016/j.nbd.2016.02.002 26850918 PMC4785038

[B49] Madsen GF, Bilenberg N, Cantio C, Oranje B (2014) Increased prepulse inhibition and sensitization of the startle reflex in autistic children. Autism Res 7:94–103. 10.1002/aur.1337 24124111

[B50] Marsden KC, Granato M (2015) In Vivo Ca(2+) imaging reveals that decreased dendritic excitability drives startle habituation. Cell Rep 13:1733–1740. 10.1016/j.celrep.2015.10.060 26655893 PMC4680997

[B51] Martineau J, Roux S, Garreau B, Adrien JL, Lelord G (1992) Unimodal and crossmodal reactivity in autism: presence of auditory evoked responses and effect of the repetition of auditory stimuli. Biol Psychiatry 31:1190–1203. 10.1016/0006-3223(92)90338-z 1391280

[B52] Martínez R, Esteve-Codina A, Herrero-Nogareda L, Ortiz-Villanueva E, Barata C, Tauler R, Raldúa D, Piña B, Navarro-Martín L (2018) Dose-dependent transcriptomic responses of zebrafish eleutheroembryos to Bisphenol A. Environ Pollut 243:988–997. 10.1016/j.envpol.2018.09.043 30248606

[B53] McAlonan GM, Daly E, Kumari V, Critchley HD, Amelsvoort T, Suckling J, Simmons A, Sigmundsson T, Greenwood K, Russell A, Schmitz N, Happe F, Howlin P, Murphy DGM (2002) Brain anatomy and sensorimotor gating in Asperger’s syndrome. Brain 125:1594–1606. 10.1093/brain/awf150 12077008

[B54] Möhrle D, Wang W, Whitehead SN, Schmid S (2021) GABAB receptor agonist R-Baclofen reverses altered auditory reactivity and filtering in the Cntnap2 knock-out rat. Front Integr Neurosci 15:710593. 10.3389/fnint.2021.710593 34489651 PMC8417788

[B55] Moy SS, Nadler JJ, Magnuson TR, Crawley JN (2006) Mouse models of autism spectrum disorders: the challenge for behavioral genetics. Am J Med Genet C Semin Med Genet 142C:40–51. 10.1002/ajmg.c.30081 16419099

[B56] Mustieles V, Pérez-Lobato R, Olea N, Fernández MF (2015) Bisphenol A: human exposure and neurobehavior. Neurotoxicology 49:174–184. 10.1016/j.neuro.2015.06.002 26121921

[B57] Nelson JC, Witze E, Ma Z, Ciocco F, Frerotte A, Randlett O, Foskett JK, Granato M (2020) Acute regulation of habituation learning via posttranslational palmitoylation. Curr Biol 30:2729–2738.e4. 10.1016/j.cub.2020.05.016 32502414 PMC8446937

[B58] Olsvik PA, Whatmore P, Penglase SJ, Skjaerven KH, Angles d’Auriac M, Ellingsen S (2019) Associations between behavioral effects of bisphenol A and DNA methylation in zebrafish embryos. Front Genet 10:184. 10.3389/fgene.2019.00184 30906313 PMC6418038

[B59] Ornitz EM, Lane SJ, Sugiyama T, de Traversay J (1993) Startle modulation studies in autism. J Autism Dev Disord 23:619–637. 10.1007/BF01046105 8106303

[B60] Perry W, Minassian A, Lopez B, Maron L, Lincoln A (2007) Sensorimotor gating deficits in adults with autism. Biol Psychiatry 61:482–486. 10.1016/j.biopsych.2005.09.025 16460695

[B61] Prut L, Belzung C (2003) The open field as a paradigm to measure the effects of drugs on anxiety-like behaviors: a review. Eur J Pharmacol 463:3–33. 10.1016/S0014-2999(03)01272-X 12600700

[B62] Qiu W, Zhao Y, Yang M, Farajzadeh M, Pan C, Wayne NL (2016) Actions of bisphenol A and bisphenol S on the reproductive neuroendocrine system during early development in zebrafish. Endocrinology 157:636–647. 10.1210/en.2015-1785 26653335

[B63] Restivo L, Ferrari F, Passino E, Sgobio C, Bock J, Oostra BA, Bagni C, Ammassari-Teule M (2005) Enriched environment promotes behavioral and morphological recovery in a mouse model for the fragile X syndrome. Proc Natl Acad Sci U S A 102:11557–11562. 10.1073/pnas.0504984102 16076950 PMC1183589

[B64] Rihel J, Prober DA, Arvanites A, Lam K, Zimmerman S, Jang S, Haggarty SJ, Kokel D, Rubin LL, Peterson RT, Schier AF (2010) Zebrafish behavioral profiling links drugs to biological targets and rest/wake regulation. Science 327:348–351. 10.1126/science.1183090 20075256 PMC2830481

[B65] Roberts AC, Reichl J, Song MY, Dearinger AD, Moridzadeh N, Lu ED, Pearce K, Esdin J, Glanzman DL (2011) Habituation of the C-start response in larval zebrafish exhibits several distinct phases and sensitivity to NMDA receptor blockade. PLoS One 6:e29132. 10.1371/journal.pone.0029132 22216183 PMC3247236

[B66] Roberts AC, Bill BR, Glanzman DL (2013) Learning and memory in zebrafish larvae. Front Neural Circuits 7:126. 10.3389/fncir.2013.00126 23935566 PMC3731533

[B67] Roberts AC, Pearce KC, Choe RC, Alzagatiti JB, Yeung AK, Bill BR, Glanzman DL (2016) Long-term habituation of the C-start escape response in zebrafish larvae. Neurobiol Learn Mem 134 [Pt B]:360–368. 10.1016/j.nlm.2016.08.014 27555232 PMC5031492

[B68] Roberts AC, Chornak J, Alzagatiti JB, Ly DT, Bill BR, Trinkeller J, Pearce KC, Choe RC, Campbell CS, Wong D, Deutsch E, Hernandez S, Glanzman DL (2019) Rapid habituation of a touch-induced escape response in zebrafish (*Danio rerio*) larvae. PLoS One 14:e0214374. 10.1371/journal.pone.0214374 30946762 PMC6449028

[B200] Roberts AC et al. (2020) Induction of short-term sensitization by an aversive chemical stimulus in zebrafish larvae. eNeuro 7:ENEURO.0336-19.2020. 10.1523/ENEURO.0336-19.2020 PMC7729299 33004417

[B69] Rochester JR (2013) Bisphenol A and human health: a review of the literature. Reprod Toxicol 42:132–155. 10.1016/j.reprotox.2013.08.008 23994667

[B70] Saili KS, Corvi MM, Weber DN, Patel AU, Das SR, Przybyla J, Anderson KA, Tanguay RL (2012) Neurodevelopmental low-dose bisphenol A exposure leads to early life-stage hyperactivity and learning deficits in adult zebrafish. Toxicology 291:83–92. 10.1016/j.tox.2011.11.001 22108044 PMC3245816

[B71] Santangeli S, Consales C, Pacchierotti F, Habibi HR, Carnevali O (2019) Transgenerational effects of BPA on female reproduction. Sci Total Environ 685:1294–1305. 10.1016/j.scitotenv.2019.06.029 31272786

[B72] Sato T, Takahoko M, Okamoto H (2006) HuC: Kaede, a useful tool to label neural morphologies in networks in vivo. Genesis 44:136–142. 10.1002/gene.20196 16496337

[B73] Schmeisser MJ, et al. (2012) Autistic-like behaviours and hyperactivity in mice lacking ProSAP1/Shank2. Nature 486:256–260. 10.1038/nature11015 22699619

[B74] Schneider CA, Rasband WS, Eliceiri KW (2012) NIH Image to ImageJ: 25 years of image analysis. Nat Methods 9:671–675. 10.1038/nmeth.2089 22930834 PMC5554542

[B75] Schnörr SJ, Steenbergen PJ, Richardson MK, Champagne DL (2012) Measuring thigmotaxis in larval zebrafish. Behav Brain Res 228:367–374. 10.1016/j.bbr.2011.12.016 22197677

[B76] Schonfelder G, Wittfoht W, Hopp H, Talsness CE, Paul M, Chahoud I (2002) Parent bisphenol A accumulation in the human maternal-fetal-placental unit. Environ Health Perspect 110:A703–A707. 10.1289/ehp.021100703 12417499 PMC1241091

[B77] Scopel CFV, Sousa C, Machado MRF, Santos WGD (2021) BPA toxicity during development of zebrafish embryo. Braz J Biol 81:437–447. 10.1590/1519-6984.230562 32490983

[B78] Singh S, Li SS (2012) Epigenetic effects of environmental chemicals bisphenol A and phthalates. Int J Mol Sci 13:10143–10153. 10.3390/ijms130810143 22949852 PMC3431850

[B79] Stein TP, Schluter MD, Steer RA, Guo L, Ming X (2015) Bisphenol A exposure in children with autism spectrum disorders. Autism Res 8:272–283. 10.1002/aur.1444 25641946 PMC4474754

[B80] Subbanna S, Nagre NN, Shivakumar M, Basavarajappa BS (2016) A single day of 5-azacytidine exposure during development induces neurodegeneration in neonatal mice and neurobehavioral deficits in adult mice. Physiol Behav 167:16–27. 10.1016/j.physbeh.2016.08.036 27594097 PMC5159185

[B81] Sundarraj S, Sujitha MV, Alphonse CRW, Kalaiarasan R, Kannan RR (2021) Bisphenol-A alters hematopoiesis through EGFR/ERK signaling to induce myeloblastic condition in zebrafish model. Sci Total Environ 787:147530. 10.1016/j.scitotenv.2021.147530 34004533

[B82] Swerdlow NR, Geyer MA (1998) Using an animal model of deficient sensorimotor gating to study the pathophysiology and new treatments of schizophrenia. Schizophr Bull 24:285–301. 10.1093/oxfordjournals.schbul.a033326 9613626

[B83] Tabor KM, Smith TS, Brown M, Bergeron SA, Briggman KL, Burgess HA (2018) Presynaptic inhibition selectively gates auditory transmission to the brainstem startle circuit. Curr Biol 28:2527–2535.e8. 10.1016/j.cub.2018.06.020 30078569 PMC6410737

[B84] Thayer KA, Doerge DR, Hunt D, Schurman SH, Twaddle NC, Churchwell MI, Garantziotis S, Kissling GE, Easterling MR, Bucher JR, Birnbaum LS (2015) Pharmacokinetics of bisphenol A in humans following a single oral administration. Environ Int 83:107–115. 10.1016/j.envint.2015.06.008 26115537 PMC4545316

[B85] Thyme SB, Pieper LM, Li EH, Pandey S, Wang Y, Morris NS, Sha C, Choi JW, Herrera KJ, Soucy ER, Zimmerman S, Randlett O, Greenwood J, McCarroll SA, Schier AF (2019) Phenotypic landscape of schizophrenia-associated genes defines candidates and their shared functions. Cell 177:478–491.e20. 10.1016/j.cell.2019.01.048 30929901 PMC6494450

[B86] Tse WK, Yeung BH, Wan HT, Wong CK (2013) Early embryogenesis in zebrafish is affected by bisphenol A exposure. Biol Open 2:466–471. 10.1242/bio.20134283 23789094 PMC3654264

[B87] Vandenberg LN, Hauser R, Marcus M, Olea N, Welshons WV (2007) Human exposure to bisphenol A (BPA). Reprod Toxicol 24:139–177. 10.1016/j.reprotox.2007.07.010 17825522

[B88] Vivanti G, Hocking DR, Fanning PAJ, Uljarevic M, Postorino V, Mazzone L, Dissanayake C (2018) Attention to novelty versus repetition: contrasting habituation profiles in autism and Williams syndrome. Dev Cogn Neurosci 29:54–60. 10.1016/j.dcn.2017.01.006 28130077 PMC6987850

[B89] Walz N, Mühlberger A, Pauli P (2016) A human open field test reveals thigmotaxis related to agoraphobic fear. Biol Psychiatry 80:390–397. 10.1016/j.biopsych.2015.12.016 26876946

[B90] Wang X, Dong Q, Chen Y, Jiang H, Xiao Q, Wang Y, Li W, Bai C, Huang C, Yang D (2013) Bisphenol A affects axonal growth, musculature and motor behavior in developing zebrafish. Aquat Toxicol 142–143:104–113. 10.1016/j.aquatox.2013.07.011 23994041

[B91] Wolman MA, Jain RA, Liss L, Granato M (2011) Chemical modulation of memory formation in larval zebrafish. Proc Natl Acad Sci U S A 108:15468–15473. 10.1073/pnas.1107156108 21876167 PMC3174630

[B92] Wolman MA, Jain RA, Marsden KC, Bell H, Skinner J, Hayer KE, Hogenesch JB, Granato M (2015) A genome-wide screen identifies PAPP-AA-mediated IGFR signaling as a novel regulator of habituation learning. Neuron 85:1200–1211. 10.1016/j.neuron.2015.02.025 25754827 PMC4368495

[B93] Wolstenholme JT, Goldsby JA, Rissman EF (2013) Transgenerational effects of prenatal bisphenol A on social recognition. Horm Behav 64:833–839. 10.1016/j.yhbeh.2013.09.007 24100195 PMC3955720

[B94] Wu CC, Shields JN, Akemann C, Meyer DN, Connell M, Baker BB, Pitts DK, Baker TR (2021) The phenotypic and transcriptomic effects of developmental exposure to nanomolar levels of estrone and bisphenol A in zebrafish. Sci Total Environ 757:143736. 10.1016/j.scitotenv.2020.143736 33243503 PMC7790172

[B95] Yamada H, Furuta I, Kato EH, Kataoka S, Usuki Y, Kobashi G, Sata F, Kishi R, Fujimoto S (2002) Maternal serum and amniotic fluid bisphenol A concentrations in the early second trimester. Reprod Toxicol 16:735–739. 10.1016/S0890-6238(02)00051-5 12401500

[B96] Yaoi T, Itoh K, Nakamura K, Ogi H, Fujiwara Y, Fushiki S (2008) Genome-wide analysis of epigenomic alterations in fetal mouse forebrain after exposure to low doses of bisphenol A. Biochem Biophys Res Commun 376:563–567. 10.1016/j.bbrc.2008.09.028 18804091

[B97] Ye X, Kuklenyik Z, Needham LL, Calafat AM (2006) Measuring environmental phenols and chlorinated organic chemicals in breast milk using automated on-line column-switching-high performance liquid chromatography-isotope dilution tandem mass spectrometry. J Chromatogr B Analyt Technol Biomed Life Sci 831:110–115. 10.1016/j.jchromb.2005.11.050 16377264

[B98] Yoo SJ, Joo H, Kim D, Lim MH, Kim E, Ha M, Kwon HJ, Paik KC, Kim KM (2020) Associations between exposure to bisphenol A and behavioral and cognitive function in children with attention-deficit/hyperactivity disorder: a case-control study. Clin Psychopharmacol Neurosci 18:261–269. 10.9758/cpn.2020.18.2.261 32329307 PMC7242102

[B99] Zoodsma JD, Chan K, Bhandiwad AA, Golann DR, Liu G, Syed SA, Napoli AJ, Burgess HA, Sirotkin HI, Wollmuth LP (2020) A model to study NMDA receptors in early nervous system development. J Neurosci 40:3631–3645. 10.1523/JNEUROSCI.3025-19.2020 32245827 PMC7189761

